# Characterization of the infectious reservoir of malaria with an agent-based model calibrated to age-stratified parasite densities and infectiousness

**DOI:** 10.1186/s12936-015-0751-y

**Published:** 2015-06-03

**Authors:** Jaline Gerardin, André Lin Ouédraogo, Kevin A McCarthy, Philip A Eckhoff, Edward A Wenger

**Affiliations:** Institute for Disease Modeling, Intellectual Ventures, 1555 132nd Ave NE, Bellevue, WA 98005 USA; Department of Biomedical Sciences, Centre National de Recherche et de Formation sur le Paludisme, Ouagadougou, Burkina Faso

**Keywords:** Mechanistic model, Infectious reservoir, Diagnostics, Infectivity, Drug campaigns

## Abstract

**Background:**

Elimination of malaria can only be achieved through removal of all vectors or complete depletion of the infectious reservoir in humans. Mechanistic models can be built to synthesize diverse observations from the field collected under a variety of conditions and subsequently used to query the infectious reservoir in great detail.

**Methods:**

The EMOD model of malaria transmission was calibrated to prevalence, incidence, asexual parasite density, gametocyte density, infection duration, and infectiousness data from nine study sites. The infectious reservoir was characterized by age and parasite detectability with diagnostics of varying sensitivity over a range of transmission intensities with and without case management and vector control. Mass screen-and-treat drug campaigns were tested for likelihood of achieving elimination.

**Results:**

The composition of the infectious reservoir is similar over a range of transmission intensities, and higher intensity settings are biased towards infections in children. Recent ramp-ups in case management and use of insecticide-treated bed nets (ITNs) reduce the infectious reservoir and shift the composition towards sub-microscopic infections. Mass campaigns with anti-malarial drugs are highly effective at interrupting transmission if deployed shortly after ITN campaigns.

**Conclusions:**

Low-density infections comprise a substantial portion of the infectious reservoir. Proper timing of vector control, seasonal variation in transmission intensity and mass drug campaigns allows lingering population immunity to help drive a region towards elimination.

**Electronic supplementary material:**

The online version of this article (doi:10.1186/s12936-015-0751-y) contains supplementary material, which is available to authorized users.

## Background

Malaria is a global disease responsible for hundreds of thousands of deaths each year [1]. In the last decade, many regions have made considerable progress in malaria control and are now working towards local elimination of malaria [2, 3]. Technical strategies for elimination differ from those for control, and it is critical to understand the factors that lead to successful outcomes and make the best use of available resources.

Elimination of malaria requires interrupting transmission. Complete depletion of the infectious reservoir of malaria parasites requires clearing all malaria infections in both human and vector populations. In regions where malaria is endemic, accumulated exposure to infection leads individuals to develop immunity to clinical symptoms and partial immunity to parasites. Proper identification of parasite carriers is therefore confounded by a population of asymptomatic people with low-density infections.

Current rapid diagnostic tests (RDT) can quickly and cheaply identify individuals with parasite densities above 50–200 parasites per μL [4]. However, in endemic areas, a significant portion of the population harbours infections that are undetectable by RDT, and gold-standard microscopy or molecular methods, both limited to research laboratories, are required to identify infected individuals. Although capable of detecting infections as low as 0.01-0.05 parasites per μL, even the most sensitive molecular tests today cannot detect all infections [5].

Asexual parasite density, gametocyte density and human infectiousness are known to be linked [6, 7]. Higher asexual densities lead to higher gametocyte densities, which increase the chance of infecting mosquitoes. However, the relationship between gametocyte density and infectiousness to mosquitoes is complex, as individuals with high gametocyte density may infect few or no mosquitoes in studies where captured mosquitoes are fed on human blood, and individuals with no observable gametocytes have been observed to be infectious [8, 9]. Human sexual-stage and transmission-blocking immunity have been proposed to play roles in moderating the relationship between gametocyte density and infectiousness [10], but the magnitude and duration of sexual stage immunity effects remain unclear.

While most malaria infections in areas of even moderate transmission are asymptomatic [11], the relative contribution to the infectious reservoir of symptomatic and asymptomatic, patent and sub-patent, and adult and child populations remains an area of active investigation [12, 13]. Sub-patent individuals, even if they outnumber patent individuals, are less infectious to mosquitoes. In an elimination setting, it is critical to understand how success may or may not depend on appropriate targeting of sub-patent infections, as sensitive diagnostics can be very resource-intensive.

Mathematical modelling of malaria transmission and immunity can begin to answer some of these questions [12, 13]. A single mechanistic model can tie together apparently contradictory field data that describe malaria transmission in a variety of settings and demographic groups. With a detailed model of within-host infection, including parasite and gametocyte life cycles and densities as well as acquired immunity, the relative contribution to the infectious reservoir due to each sub-set of the population can be compared and implications for control and elimination can be quantitatively assessed.

A recent longitudinal study in a high-transmission area of Burkina Faso used highly sensitive molecular detection methods to quantify asexual and gametocyte densities, providing invaluable data for model calibration [14] ethics approval No. 2007-035/MS/MESSRS/CERS and 2014-0058/MS/MERSI/CERS. This study also measured infectiousness to mosquitoes with membrane feeding tests where 50 mosquitoes were fed on blood from each study participant and subsequently dissected to determine oocyst counts. Using this dataset and an agent-based stochastic model of malaria transmission, adaptive immunity parameters were calibrated to best capture the age-dependent distribution of asexual parasite and gametocyte densities. Next, oocyst positivity from the membrane feeding data was used to calibrate model parameters governing human infectiousness to mosquitoes. The calibrated model was then used to characterize the composition of the infectious reservoir by detection threshold and age group through seasonal variations and at a range of transmission intensities. Changes in the infectious reservoir after deployment of case management and vector control are described. Finally, the implications for elimination campaigns are discussed by testing how mass drug campaigns may successfully achieve elimination by targeting all or only a portion of the infectious reservoir.

## Methods

### Malaria transmission model

All simulations were conducted with EMOD DTK v1.6, an agent-based mechanistic model of malaria transmission [15]. During the blood stage of malaria infection, asexual parasite density triggers innate and adaptive immunity within the host. Innate immunity stimulates cytokine production, leading to fever in a density-dependent manner and limiting the maximum parasite density. Adaptive immunity is modelled using three types of antigens: *Plasmodium falciparum* erythrocyte membrane protein 1 (PfEMP1) variants, merozoite surface proteins (MSP), and minor epitope variants. Fuller discussion of immune system modelling within the EMOD model is described in [16–18]. Asexual parasite and gametocyte densities are tracked within each host. Gametocytes differentiate from infected red blood cells and mature in five stages over ten days, and a fraction of gametocytes are lost at each stage of maturation from implicit immune effects. Mature stage gametocytes are taken up in a mosquito blood meal with density-dependent probability, and human and mosquito immune factors implicitly limit gametocyte survival within the mosquito. Left untreated, all asexual and gametocyte stage parasites will eventually be cleared by host immune factors. Infectiousness of an individual human is calculated as a xenodiagnostic experiment measuring the fraction of mosquitoes infected after feeding on the individual.

### Calibration of asexual parasite and gametocyte densities by age and season

Calibration of parasite densities built on previous work in [19]. Data from nine study sites were used, including four sites with age-stratified prevalence data: Namawala in Tanzania and Matsari, Rafin Marke and Sugungum in Nigeria, and two sites with age-stratified clinical incidence data: Dielmo and Ndiop in Senegal, that span annual entomological inoculation rates (EIRs) between 18 and over 300 and have been used in previous work [20–23]. Recently available data from two sites, Dapelogo and Laye in Burkina Faso, provided age- and season-stratified asexual parasite and gametocyte densities as measured by molecular methods [14]. Malariatherapy data on peak parasitaemia and gametocytaemia as well as duration of parasitaemia and gametocytaemia for single infections in naïve adults were also included in the calibration [24].

Parameters under calibration included the maximum number of simultaneous infections, number of PfEMP1 variants available to the parasite population, switching rate between PfEMP1 variants, number of MSP variants, number of minor epitope variants, killing strengths of anti-MSP and anti-minor epitope immune responses, production rate of gametocytes, and survival rate of gametocytes as they progress through stages of maturation.

Prevalence and incidence sites were simulated and likelihoods of calibration parameters were calculated as described in [19]. Simulated individuals in endemic study sites were subjected to an age- and month-dependent force of infection. Monthly EIR for Dapelogo and Laye followed Burkina Faso seasonality and were based on available entomological data, with an annual EIR of 300 for Dapelogo and 30 for Laye [25]. Malariatherapy simulations included a single infectious bite on day 0 with no other infectious bites.

For malariatherapy simulations, asexual parasite and gametocyte densities were measured daily in 1000 naïve adults. For all other calibration study sites, a birth cohort of 1000 children was followed for 100 years, and incidence and prevalence measurements were gathered monthly and annually to compare with field data.

**Fig. 1:**
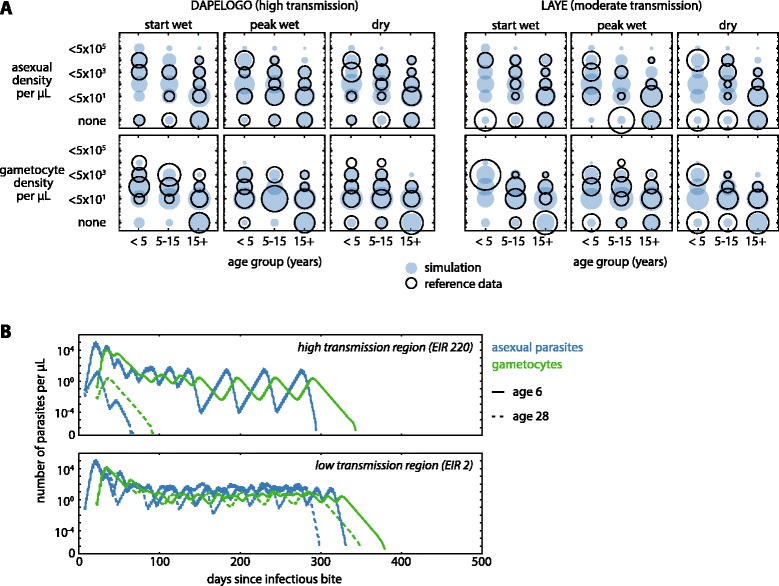
Calibration of asexual parasite and gametocyte densities. **a** The distribution of asexual parasite and gametocyte densities by season and age group in reference data (open circles) and post-calibration simulation (filled circles) is shown for two study sites. Circle area is proportional to number of individuals and normalized for each season of each age group. **b** Example infection trajectories of asexual parasites (blue) and gametocytes (green) after calibration for an age-6 child (solid lines) and age-28 adult (dotted lines) with immune histories corresponding to high-transmission (top) and low-transmission (bottom) settings. Each individual was challenged with a single infectious bite on day 0, with no subsequent biting. No drugs were administered and host immunity eventually cleared all parasites

In the likelihood function used to calibrate asexual parasite and gametocyte densities, data from each age group (under five, five to 15, over 15), season (start of wet season, peak of wet season, dry season), study site (Dapelogo, Laye), and parasite stage (asexual, gametocyte) were considered separately (Fig. 1). Asexual parasite and gametocyte densities were collected in simulations on 1 July (start of wet season), 1 September (peak of wet season), and 1 March (dry season), binned for each age group, and compared with field data with a Dirichlet-multinomial distribution.

For each parameter set $\overrightarrow{\theta}$, the parasite/gametocyte density distributions in each age group, site, and season are binned into six density bins. The likelihood of parameter set $\overrightarrow{\theta}$ given the data *d* (consisting of *n*_*d*_ total measurements, $\overrightarrow{k_d}$ counts in each bin, ${\displaystyle \sum_{i=1}^6}{k}_{d,\ i}={n}_d$) is approximated as follows. The prior distribution for the multinomial probability vector $\overrightarrow{p}$ associated with the six bins is initially assumed to be a symmetric Dirichlet distribution with concentration parameter 1. The simulated distribution of counts $\overrightarrow{k_s}$ is used to inform the posterior distribution of $\overrightarrow{p}$:

$$ Dir\left(\overrightarrow{p}\Big|\overrightarrow{1}\right)\ \to\ Dir\left(\overrightarrow{p}\Big|\overrightarrow{1} + \overrightarrow{k_s}\right) $$

The Dirichlet posterior in the likelihood function reflects the degree of uncertainty on the true $\overrightarrow{p}$ at a particular parameter set, given stochastic fluctuations in the observed $\overrightarrow{k_s}$ and a uniform prior $Dir\left(\overrightarrow{p}\Big|\overrightarrow{1}\right)$. The likelihood of $\overrightarrow{\theta}$ is then approximated using the posterior predictive distribution computed by marginalizing over $\overrightarrow{p}$:

$$ \mathrm{\mathcal{L}}\left(\overrightarrow{\theta}\Big|d\right) = P\left(d\Big|\overrightarrow{\theta}\right) = {\displaystyle \int }P\left(d\Big|\overrightarrow{p}\right)P\left(\overrightarrow{p}\Big|\overrightarrow{\theta}\right)d\overrightarrow{p}={\displaystyle \int } Mult\left(\overrightarrow{k_d}\Big|\ {n}_d,\ \overrightarrow{p}\right)Dir\left(\overrightarrow{p}\Big|\overrightarrow{1} + \overrightarrow{k_s}\right)d\overrightarrow{p} = DirMult\left(\overrightarrow{k_d}\Big|{n}_d,\ \overrightarrow{1} + \overrightarrow{k_s}\right) $$

The joint likelihood is the product of likelihoods for each age group, season, study site, and parasite stage, which is also multiplied with the likelihoods from prevalence, incidence and malariatherapy comparisons to determine the overall likelihood of each parameter set. Likelihoods for prevalence and incidence are approximated using a methodology similar to that described above (detailed in [19]), using beta-binomial and gamma-Poisson distributions, respectively.

For calibration to malariatherapy data, patients who received anti-malarial treatment were removed from the comparison data. All patients were assumed to have only a single infection. Distributions of peak parasitaemia, peak gametocytaemia, parasitaemia duration, and gametocytaemia durations were compared to simulation using a Dirichlet-multinomial likelihood as described above.

Parameter sampling was done by incremental mixture importance sampling (IMIS), as described in [26]. Final best-fit parameter values are shown in Additional files 1 and 2 and comparison with study site data shown in Additional file 3. Malariatherapy data were weighted at 10 % relative to data from endemic areas in the likelihood function, as full weighting of malariatherapy data resulted in poorer fitting to prevalence data, particularly at the Rafin Marke study site (in Additional file 4). Infections from malariatherapy studies appear to have relatively short durations compared to what would be expected based on data collected from endemic sites. The malariatherapy dataset used in calibration, which did not include any patients who received curative treatment with drugs, may be biased towards milder infections. Strain differences between parasites used for malariatherapy and parasites in endemic regions may also account for the discrepancies. Differences in infection duration between strains have been noted previously, with El Limon showing long infections, McLendon showing short infections, and Santee Cooper showing both long and short infections [18].

### Calibration of infectiousness by age and season

Human infectiousness to mosquitoes was calibrated to membrane feeding data stratified by age, season and study site [14]. Simulations used the immunity, infection and gametocyte development parameters in Additional file 1. A population of 1000 individuals of all ages was subjected to 50 years of forced EIR to initialize age-appropriate immunity, with EIR values as described above for the Dapelogo and Laye sites. Calibration also used forced EIR and no vectors.

**Fig. 2:**
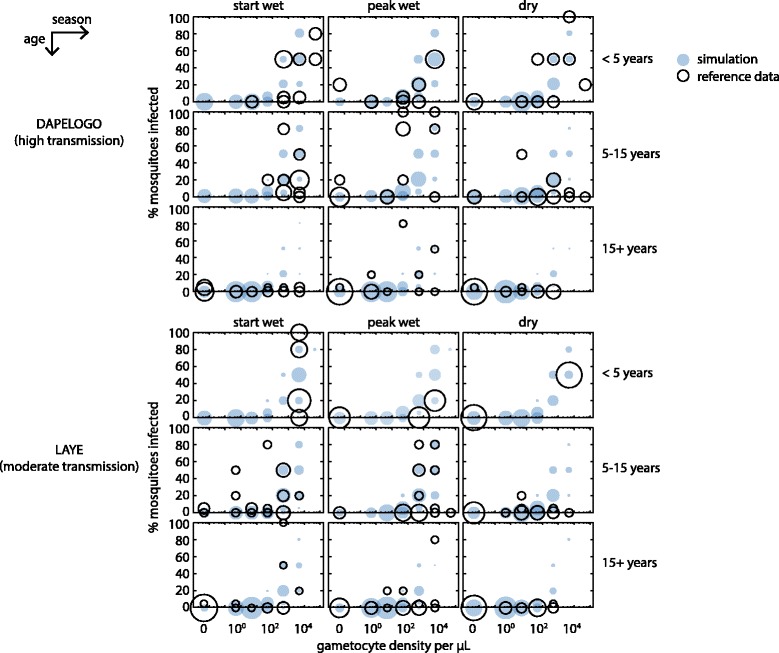
Calibration of human infectiousness to mosquitoes. The relationship between gametocyte density and infectiousness by season and age group in reference data (open circles) and post-calibration simulation (filled circles) is shown for two study sites. Circle area is proportional to number of individuals and normalized for each season of each age group

Two parameters, the survival rate of gametocytes in the mosquito and the maximum probability a mosquito becomes infected, were calibrated using IMIS as described above. For each sampled parameter set, the distribution of fraction mosquitoes infected was compared to field data with a Dirichlet-multinomial as described above. Infectiousness by age group (under five, five to 15, over 15) and season (start of wet season, peak of wet season, dry season) were considered separately, and likelihoods were multiplied. Calibrated parameter values are shown in in Additional file 2 and simulated infectiousness compared with field data in Fig. 2.

### Characterization of the infectious reservoir in full vector transmission model

Simulations of the infectious reservoir were conducted with a full vector transmission model in a Burkina Faso climate with vector abundance calibrated to achieve annual EIR between 200 and 300. Vector species included *Anopheles gambiae* and a small amount of *Anopheles funestus*, and larval habitats were modelled as in [27] with enough constantly available habitat to ensure vector survival through the dry season. Vector feeding behaviour was 95 % anthropophagic and 95 % endophagic; other vector life cycle parameters were modelled as in [27]. Population demographics followed the Burkina Faso age structure [28], including birth and age-dependent mortality. All simulations were performed on a well-mixed population of 1000 individuals. Larval habitat was scaled to simulate settings with lower transmission intensity (Additional file 5). Simulation of each transmission intensity was repeated for 1000 stochastic realizations, and all figures show mean measurement values. Populations were simulated for 50 years at each transmission intensity in the absence of interventions to initialize age-appropriate immunity. Importation of cases occurred at a rate of one per year. All simulations included one year of equilibration prior to three years of measurement.

Individuals were assigned to detection groups on each day of the detection period. Each individual was tested for asexual parasite positivity at three diagnostic sensitivity levels: 100, 10 and 0.05 asexual parasites per μL and assigned to the least sensitive diagnostic to which they tested positive. Individuals tested positive if a random draw from a Poisson distribution centred at the true number of asexual parasites in 1/(diagnostic sensitivity) μL of blood was non-zero. Individuals testing negative by all diagnostics, but nonetheless infected with asexual parasites, were assigned to the undetectable group.

Host size is known to predict mosquito biting behaviour, and an age-dependent biting risk was included to approximate surface area dependence [29–31]. Composition of the infectious reservoir is calculated by normalizing the scaled human infectiousness. Uncertainty in parameter values and stochastic variations between simulation runs does not qualitatively change the composition of the infectious reservoir (Additional file 6).

### Modelling case management and campaigns with insecticide-treated bed nets (ITNs)

Several simulation scenarios are modelled: baseline ‘no intervention’ scenarios with no case management or ITNs; case management scenarios where symptomatic individuals could access treatment with anti-malarial drugs, but no ITNs; ITN campaign scenarios where ITNs were distributed to the population in an age-dependent manner, but no case management was available; and, scenarios that included both ITN distribution and case management.

Case management was available to simulated individuals with clinical or severe malaria. A clinical case occurred when an individual’s body temperature increased by at least 1.5 °C, and severe cases occurred when fever, anaemia or parasite density exceeded thresholds described in [19]. Ninety-five per cent of the population had any access to case management. Of the population with access to case management, individuals with clinical malaria had 60 % probability of seeking care for a given episode. Individuals with severe malaria had 95 % probability of seeking care. All individuals seeking care received treatment with artemether-lumefantrine (AL) within three days for clinical cases or two days for severe cases, and all treated individuals completed the full course of treatment with full adherence. Age-dependent dosing, pharmacokinetics and pharmacodynamics of AL were modelled as described in [32]. Simulations with case management allowed case management during both the equilibration and measurement periods.

ITNs were distributed at birth with 90 % coverage and were also mass distributed on day 200 of the first measurement year with 80 % coverage for children under ten and 50 % for individuals over ten years of age. ITNs had an initial blocking rate of 90 % and killing rate of 60 %, with efficacy decaying exponentially with half-lives of two years and four years, respectively. When ITNs were present, the contribution of ITN-protected individuals to the infectious reservoir was reduced by the ITN blocking rate after scaling by surface area as discussed above.

Annual EIR was measured by summing the daily infectious bites per person over the second measurement year. For the same amount of larval habitat, case management and ITNs reduced the apparent EIR relative to simulations without interventions. When case management and/or ITNs were present, the plotted EIR is the apparent EIR experienced by the simulated population.

### Simulation of mass drug campaigns

Mass drug campaigns were conducted with dihydroartemisinin-piperaquine (DP), with improved paediatric dosing as described in [33] and pharmacokinetics and pharmacodynamics as in [32]. All individuals receiving DP adhered to the full treatment course. Mass campaigns were conducted during the second and third measurement years in three rounds per year separated by six weeks, with the first round occurring on day 60 of the second measurement year. All covered individuals received DP on the same day. Coverage was independent between rounds. Importation of cases occurred at a rate of one per year.

Mass campaigns were tested at apparent EIRs between 0.07 and 40 for settings with no other interventions and settings with case management and ITN use as described above. Drug campaign coverage was tested at 0, 20, 40, 60, 80, and 100 %. Interpolations were calculated with the SciPy v0.14.1 interpolate function in Python 2.7. Mass screen-and-treat (MSAT) campaigns used diagnostics with sensitivity 100, 20 or 2 asexual parasites per μL. Simulation of each EIR, coverage and MSAT diagnostic sensitivity was repeated for 100 stochastic realizations.

Elimination was determined to be achieved if true asexual parasite prevalence was 0 for the last 150 days of simulation year 3. Probability of elimination was the fraction of the 100 stochastic realizations resulting in elimination.

## Results

### Composition of the infectious reservoir in the absence of interventions

Adaptive immunity and human infectiousness parameters within an agent-based model were calibrated to age- and season-stratified data from two high-transmission areas in Burkina Faso (Figs. 1 and 2) (see Methods). Typical infection trajectories in the calibrated model show shorter durations of infection and lower parasite densities in adults, especially in high-transmission settings (Fig. 1b and Additional file 7). Gametocyte clearance lagged behind asexual parasite clearance, and all infections were eventually cleared by host immunity.

**Fig. 3:**
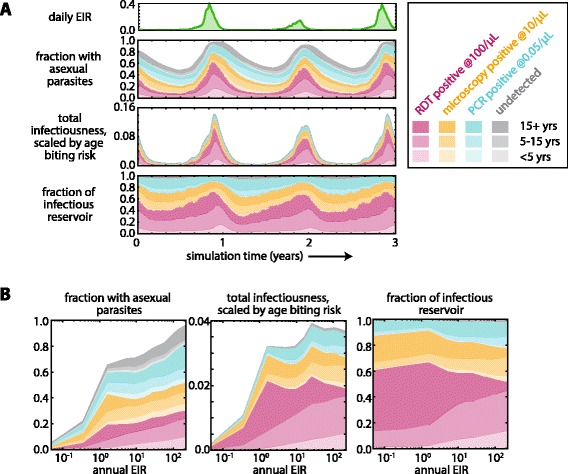
Composition of the infectious reservoir in the absence of interventions.**a** The composition of the infectious reservoir by diagnostic detectability and age in a setting with moderate transmission. A region of EIR = 10 was simulated over three years with seasonal rainfall and temperature based on Burkina Faso climate. The entire population was tested for asexual parasite prevalence daily for three years with diagnostics at three levels of sensitivity: 100 parasites/μL (bottom 3 stripes, pink), 10 parasites/μL (3 stripes second from bottom, yellow), and 0.05 parasites/μL (3 stripes third from bottom, blue). Infections undetectable at 0.05 parasites/μL are shown in grey (top 3 stripes). Infectiousness was calculated by simulating a membrane-feeding test and subsequently scaling by age to approximate surface area effects. The fraction of the infectious reservoir is the normalization of total infectiousness. The average of 100 stochastic realizations is shown. **b** Annual average composition of the infectious reservoir under a range of transmission intensities. All measurements were averaged over the second year of simulation. 1000 stochastic realizations were averaged for each EIR level

Figure 3a shows the infectious reservoir over three years in a moderate-transmission setting with annual EIR of 10 and the seasonality of Burkina Faso. Total asexual parasite prevalence peaks towards the end of the rainy season and is largely due to a large increase in higher-density infections. Prevalence of RDT-negative, microscopy-positive infections as well as infections positive by PCR only and undetectable infections remained relatively more constant through the year, peaking towards the beginning of the dry season as the RDT-positive infections began to clear. For this work, the limit of detection for microscopy is considered to be the gold-standard 10 parasites/μL achievable in research facilities rather than the 100 parasites/μL more common in the field.

Total infectiousness was quantified by measuring the probability of each individual infecting a mosquito and scaling by age to approximate the decreased biting risk of children due to smaller surface area (see Methods). Total infectiousness exhibits more seasonal variation than asexual parasite prevalence since transmission to mosquitoes is dominated by RDT-positive individuals, and higher parasite density leads to greater infectiousness.

Under these conditions of moderate transmission and high seasonality, the composition of the infectious reservoir shows considerable variation through the year. RDT-positive individuals comprise over 60 % of the infectious reservoir during the wet season but only 30 % during the dry season. Sub-microscopic infections account for 20-40 % of the infectious reservoir at any time of year, a substantial portion.

While children have higher parasite densities and are highly infectious (Figs. 1 and 2), their smaller surface area reduces their contribution to the total infectiousness of the population. Children contribute the most to the infectious reservoir at the beginning of the dry season when adult immune systems have already reduced parasite load in adults, leaving children to slowly clear their longer infections and continue transmission (Fig. 1b).

To compare the infectious reservoir across a range of transmission intensities, the seasonal variation in asexual parasite prevalence and total infectiousness were averaged over a year of simulation (Fig. 3b). Total asexual parasite prevalence increases steeply as EIR increases from <0.1 to 1 infectious bite per person per year. Above EIR = 1, asexual parasite prevalence increases more gradually and approaches 100 % for EIR >100. Total population infectiousness also increases steeply as EIR increases towards 1 but is nearly constant for EIRs between 1 and 200, consistent with observations in a study area where the proportion of infected mosquitoes remained roughly constant while slide prevalence in humans declined nearly four-fold [34].

RDT-detectable infections comprise 20-30 % of all infections over the entire sampled range of transmission intensities, with higher EIR settings showing relatively more RDT-detectable infections (Additional file 8). These observations are consistent with the proposed hypothesis that individuals in high-transmission settings are constantly subjected to bursts of high parasite density from new infections, while individuals in low-transmission settings experience a long period of low-density infection as untreated single infections are cleared by immune activity [12].

RDT-detectable infections represent a similar proportion of the infectious reservoir regardless of transmission intensity, as do microscopy-detectable and PCR-detectable infections. Populations in regions with very low transmission intensity (EIR <1) have slightly more of the infectious reservoir contributed from RDT-positive individuals, while RDT-negative individuals form an increasingly large portion of the reservoir as EIR increases above 1.

Children contribute more to the infectious reservoir at the highest transmission intensities (EIR >100). At high transmission intensity, adult immunity retains memory of a large repertoire of antigenic variants, and therefore parasite density is unlikely to remain above the RDT detection limit for long. In this situation, children comprise a larger portion of the RDT-positive population. At low transmission (EIR <1), children under five barely contribute to the infectious reservoir, and children between five and 15 contribute only 20 %. Adults are the major drivers of transmission in all but the highest transmission settings.

### Composition of the infectious reservoir after recent ramp-up in case management and ITN use

Regions considering elimination are likely to have already implemented vector control and strengthened their health systems. Aggressive case management and campaigns with ITNs on the infectious reservoir were modelled. Both case management and ITN campaigns decreased the observed EIR (Additional file 9).

**Fig. 4:**
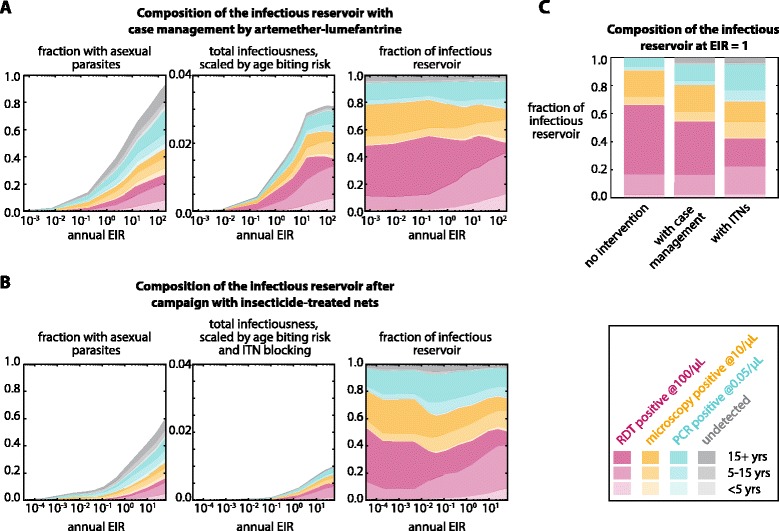
Composition of the infectious reservoir by diagnostic detectability and age with case management and deployment of insecticide-treated nets (ITNs) under a range of transmission intensities. **a** The infectious reservoir under a range of transmission intensities when case management is available. All treated individuals received a full course of artemether-lumefantrine (AL) with age-dependent dosing. See Methods for details of treatment availability and conditions for seeking care. **b** The infectious reservoir under a range of transmission intensities after deployment of ITNs. See Methods for details of ITN distribution and effectiveness. Scaling human infectiousness for age biting risk included effects of net usage. **c** Comparison of the infectious reservoir at apparent EIR of 1 under conditions of no intervention, case management with AL and ITN use

Under case management with AL, where 95 % of the population has access to care, 60 % of clinical cases seek treatment and 95 % of severe cases seek treatment, the total infectious reservoir is reduced (Fig. 4a) compared to simulations without case management (Fig. 3b). At low transmission (EIR <1), case management is particularly effective at depleting the infectious reservoir, as infections are more likely to become clinical cases and treatment of each case may clear a significant portion of the infectious reservoir.

An ITN campaign was simulated with ITN use skewed towards children (see Methods), as has been recommended for control situations [35]. Each individual’s infectiousness was scaled by the ITN blocking rate if the individual received an ITN in the simulation. Because ITNs interrupt transmission by killing vectors and blocking feeds, deployment of ITNs substantially reduces the infectious reservoir at all transmission levels (Fig. 4b). Children under five comprise an even smaller portion of the infectious reservoir than the case with no ITNs, even at high transmission.

Relative to no interventions, high case management and ITN usage decrease the overall infectiousness of the population and bias the infectious reservoir towards RDT-negative and sub-microscopic infections, especially for low-transmission settings. Case management and ITN use, which are biased towards children, further shift the infectious reservoir towards adults. At the same observed annual EIR of 1, settings where EIR has recently been reduced by case management or ITNs show a larger fraction of the infectious reservoir stemming from sub-patent infections relative to settings where EIR has historically been at 1 (Fig. 4c). Settings where EIR has recently been reduced to 1 have populations whose immune systems are adapted to EIR >1, so quickly reducing EIR leads to infections with lower parasite density. If the reduced EIR is maintained for many years, population immunity will rebound [36], and the composition of the infectious reservoir will lean more towards RDT-positive individuals.

### Depletion of the infectious reservoir after mass drug campaigns

**Fig. 5:**
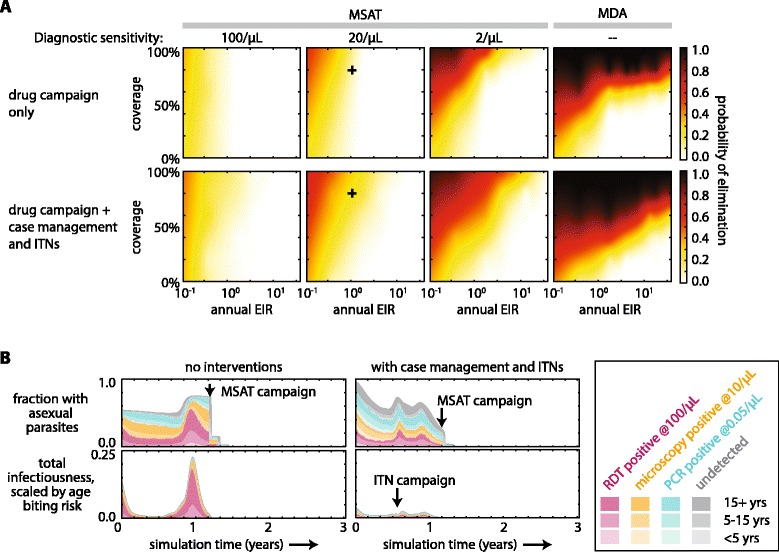
Depletion of the infectious reservoir after mass drug campaigns with dihydroartemisinin-piperaquine (DP). **a** Probability of elimination after two consecutive years of mass drug campaigns at three rounds per year. See Methods for details of timing of campaign rounds. Case management and ITNs were simulated as in Fig. 4. Probability of elimination was the fraction of 100 stochastic realizations resulting in complete elimination of all parasites by the end of year 3. Coverage was independent for all rounds and all interventions. For simulations with case management and ITNs, EIR is the EIR that would have been experienced during the second year of simulation had the drug campaigns not been administered. Crosses indicate the EIR, coverage and MSAT diagnostic sensitivity simulated in panel B. **b** The infectious reservoir before, during and after MSAT campaigns in areas with apparent EIR = 1, MSAT diagnostic sensitivity 20/μL, and 80 % coverage. Left: asexual parasite prevalence and human infectiousness in an endemic region with EIR 1 and no interventions. Right: asexual parasite prevalence and human infectiousness in a region where case management and ITN campaigns have reduced EIR to 1

The probability of elimination following MSAT campaigns with DP was tested at various levels of MSAT diagnostic sensitivity, coverage and transmission intensity (Fig. 5a). Three rounds of MSAT with independent coverage were applied during the dry season for two years. An improved paediatric formulation of DP was administered to avoid under-dosing in young children [33, 37]. The MSAT campaign outcomes were compared to mass drug administration (MDA), where all individuals are treated, as well as MSAT campaigns where case management and ITNs have recently reduced EIR.

High coverage, high MSAT diagnostic sensitivity and low transmission all increase the likelihood of elimination following an MSAT campaign. Higher coverage cannot completely compensate for lower diagnostic sensitivity, and elimination is not possible for settings with EIR >1 when an MSAT is conducted with current RDTs, which have sensitivity around 100 parasites per μL. A field trial of an MSAT campaign in a Burkina Faso site with asexual parasite prevalence between 30 and 50 % showed little success with long-term prevalence reduction [38].

Case management and ITN use increase the likelihood of elimination by MSAT. Under the same transmission intensity, lingering population immunity helps push a region towards elimination after a drug campaign if EIR has recently been reduced (Fig. 5b). The bonus from lingering immunity increases with better coverage, underscoring the critical importance of treating as many people as possible in a drug campaign. Simulation results also suggest that lingering immunity may be most beneficial for elimination efforts at EIR between 1 and 10, where EIR is low enough that elimination is possible but high enough that adult immunity is strong.

## Discussion

To interrupt the chain of malaria transmission, it is critical to identify who is transmitting. Here a mechanistic model of within-host parasite and immune dynamics was calibrated to field data on gametocyte densities and transmission probabilities. The model was then used to predict the infectious reservoir of malaria by age group and parasite detectability over a wide range of transmission intensities.

While high-quality field data is essential for understanding malaria transmission, models are also invaluable for showing that data from diverse geographic, demographic and intervention conditions can arise from the same underlying mechanisms. In this work, malariatherapy data on infection duration is incompatible with prevalence measurements from endemic areas in the EMOD model framework, highlighting a likely area for model improvement.

Because the EMOD model was calibrated to data from endemic areas of moderate to high transmission, extrapolations to low-transmission simulations are less certain, especially in light of the calibrated parameterization’s differences from malariatherapy data. Measurements of average annual parasite prevalence from endemic areas predict infections to last longer than is observed in malariatherapy patients. It is possible the model is operating in a sub-optimal regime of *P. falciparum* antigenic variants for reasonable predictions of the infectious reservoir at EIR = 0.1, as strain diversity can be much lower in low-transmission settings [39]. Long-term exposure to a few strains could lead to populations with many low-density infections and very few clinical cases, biasing the infectious reservoir towards less detectable infections. New knowledge of parasite prevalence, densities and infectiousness in low-transmission study sites will be incorporated into the model as data becomes available.

While adaptive immunity towards asexual parasites is fairly well understood, it remains unclear how sexual stage immunity affects gametocyte production, survival within the host and ability to continue the parasite life cycle within the vector [10, 25, 40–42]. The EMOD model of host immunity did not include any immunity towards gametocytes, and the trend of lower gametocyte density with age is due entirely to lower asexual parasite densities. Comparison of calibrated simulations of gametocyte density to observed distributions of gametocyte densities does not suggest that sexual stage immunity plays a strong role, at least in the high-transmission settings where the data were collected.

Transmission-blocking immunity, where individuals with high gametocyte counts fail to infect a large portion of feeding mosquitoes, has also been proposed. Calibration of the EMOD model suggests that transmission-blocking immunity may exist, as the model systematically failed to replicate data where individuals with gametocyte densities >1000/μL were sometimes observed to infect <5 % of mosquitoes. The model also does not account for cases where individuals with very low or undetectable parasite density have been observed to infect mosquitoes [43]. Thus the contribution to the infectious reservoir of people with high-density infections may have been overestimated, implying that RDT-negative infections are even more critical to target than the analysis suggests. Measurement uncertainty of molecular methods could also play a role in overestimation of gametocyte densities [44]. In addition, direct skin feeding is known to infect mosquitoes at a higher rate than membrane feeds [45]. Future data collection on transmission by direct skin feeding or concentration of mature gametocytes in the skin will be invaluable for improving models of malaria transmission and understanding the nature of the infectious reservoir.

Simulations show that children comprise a large portion of the infectious reservoir only at very high transmission intensity, and adults (aged over 15 years in the simulations) are the main drivers of transmission in low to moderate transmission settings. These results are for annual averages, but the infectious reservoir also varies seasonally, and relative contribution from children increases at the end of the wet season when adult infections have largely cleared. The age structure of the infectious reservoir will also change if mobile adults are re-importing infections from another setting where transmission is less seasonal or if one age group is spending more time outdoors in an area where transmission by outdoor-biting vectors is significant. Local patterns of behaviour and entomology can lead to region-specific patterns of exposure and thus region-specific infectious reservoirs.

Based on warnings that paediatric dosing of DP is insufficient, dosage of DP in the simulations was increased over current recommended levels. Drug campaign outcomes improved compared to previous work, which followed current guidelines for dosing, particularly in increasing the probability of elimination at EIR >10 at moderately high coverage levels [32]. While correct dosing is important for reducing the chance of recrudescence in individual patients, under-dosing is particularly critical to avoid in an elimination scenario, as interrupting transmission cannot occur when a sub-group of the population can continue to harbour and transmit infection or when a sub-group does not receive the benefits of prophylaxis.

Compared to settings where no interventions have perturbed the EIR for a long period, settings where EIR has recently been reduced show a shift in the infectious reservoir towards younger individuals and towards lower density infections. This may suggest that MSAT campaigns should achieve lower success rates in settings with recently reduced EIR because diagnostics will miss a greater fraction of the infectious reservoir. However, simulations show that MSAT campaigns are actually more successful in settings with recently reduced EIR because strong population immunity more than compensates for ongoing transmission from low-density infections.

Simulations of MSAT campaigns predict that proper timing of drug campaigns relative to ITN deployment may be critical to harness the power of lingering immunity in order to drive the region towards elimination. However, there is a lack of data to properly calibrate the rate of immunity decay, and predictions of likely elimination may be optimistic. While gathering such data may prove extremely challenging, a thorough understanding of immunity decay is critical for meaningful modelling of elimination scenarios.

## Conclusions

Composition of the infectious reservoir varies seasonally, with higher density infections forming a larger portion during the high-transmission season. RDT-negative infections make up a substantial portion of the infectious reservoir over a wide range of transmission intensities. The increased infectiousness of children due to higher gametocyte densities is balanced by decreased propensity to be bitten due to smaller surface area. Adults comprise the largest fraction of the infectious reservoir at low to moderate transmission intensities, while children form the largest portion only in very high transmission settings.

Interventions such as case management and ITN use tilt the infectious reservoir towards sub-microscopic infections. Mass campaigns with anti-malarial drugs are more successful when they reach a larger portion of the infectious reservoir through more sensitive diagnostics or higher coverage. Proper timing of drug campaigns with seasonal variation in transmission intensity as well as recent deployment of ITNs allows lingering population immunity to help drive a region towards elimination.

## Additional files

Additional file 1:
**Parameter values chosen after calibration to prevalence, incidence, and density data.**
Additional file 2:
**Parameter values chosen after calibration to infectiousness data.**
Additional file 3:
**Comparison of incidence, prevalence, peak density, and infection duration between reference data and simulation with calibrated immunity and gametocyte development parameters.**
Additional file 4:
**Comparison between simulation and reference data after calibration of immune and gametocyte parameters with full weighting of malariatherapy data.**
Additional file 5:
**Apparent EIRs tuned by scaling available larval habitat.**
Additional file 6:
**Sensitivity of the composition of the infectious reservoir to stochastic variation and uncertainty in parameter values for high- and moderate-transmission settings by age group and asexual parasite detection limit.**
Additional file 7:
**Trajectories of infection for children and adults in high- and low-transmission settings with calibrated immune and gametocyte parameters.**
Additional file 8:
**Unscaled total infectiousness by diagnostic threshold and age group.**
Additional file 9:
**Malaria transmission under case management and ITN use.**
