# Supplementary material for: Characterization of the infectious reservoir of malaria with an agent-based model calibrated to age-stratified parasite densities and infectiousness
Source: Malar J. 2015 Jun 3;14:231. doi: 10.1186/s12936-015-0751-y (PMC4702301; doi:10.1186/s12936-015-0751-y)
Supplement: Additional file 1: — Parameter values chosen after calibration to prevalence, incidence, and density data. [file 12936_2015_751_MOESM1_ESM.docx]

Parameter values chosen after calibration to prevalence, incidence, and density data. Range corresponds to the observed range in each parameter in the 100 highest-likelihood parameter sets.

| Parameter name | Parameter description | Parameter value (range) |
| --- | --- | --- |
| Max_Individual_Infections | Limit on the number of infections an individual can have simultaneously | 3 (3, 3) |
| Antigen_Switch_Rate | Antigenic switching rate per infected red blood cell (IRBC) per asexual cycle | 2.96e-9 (1.66e-9, 6.44e-9) |
| Falciparum_PfEMP1_Variants | The number of distinct PfEMP1 variants for *P. falciparum* malaria in the overall parasite population in the simulation | 1112 (1055, 1206) |
| Falciparum_MSP_Variants | The number of distinct MSP variants for *P. falciparum* malaria in the overall parasite population in the simulation | 7 (5, 11) |
| MSP1_Merozoite_Kill_Fraction | Fraction of merozoites inhibited from invading new erythrocytes when MSP1-specific antibody level is at maximum | 0.43 (0.40, 0.47) |
| Falciparum_Nonspecific_Types | The number of distinct *P. falciparum* minor epitope variants in the overall parasite population in the simulation | 90 (80, 99) |
| Nonspecific_Antigenicity_Factor | Scales antibody IRBC kill rate (set by the EMOD Antibody_IRBC_Kill_Rate parameter, which is not under calibration) for antibody responses to antigenically weak surface proteins (minor epitopes) | 0.42 (0.38, 0.50) |
| Base_Gametocyte_Production_Rate | Fraction of IRBCs producing gametocytes | 0.044 (0.029, 0.092) |
| Gametocyte_Stage_Survival_Rate | Rate of gametocyte survival from one development stage to the next in the absence of drugs or inflammatory immune response | 0.82 (0.76, 0.88) |
