# Supplementary material for: Characterization of the infectious reservoir of malaria with an agent-based model calibrated to age-stratified parasite densities and infectiousness
Source: Malar J. 2015 Jun 3;14:231. doi: 10.1186/s12936-015-0751-y (PMC4702301; doi:10.1186/s12936-015-0751-y)
Supplement: Additional file 2: — Parameter values chosen after calibration to infectiousness data. [file 12936_2015_751_MOESM2_ESM.docx]

Parameter values chosen after calibration to infectiousness data. Range corresponds to the observed range in each parameter in the 100 highest-likelihood parameter sets.

| Parameter name | Parameter description | Parameter value (range) |
| --- | --- | --- |
| Base_Gametocyte_Mosquito_Survival_Rate | Average fraction of gametocytes in blood meal that are successful in infecting a mosquito in the absence of other modulating effects, such as fever | 0.00088 (0.00013, 0.061) |
| Acquire_Modifier (vector species parameter) | Caps the probability of successful infection of a mosquito by a malaria-infected individual | 0.8 (0.61, 0.94) |
