# Supplementary material for: Characterization of the infectious reservoir of malaria with an agent-based model calibrated to age-stratified parasite densities and infectiousness
Source: Malar J. 2015 Jun 3;14:231. doi: 10.1186/s12936-015-0751-y (PMC4702301; doi:10.1186/s12936-015-0751-y)
Supplement: Additional file 3: — Comparison of incidence, prevalence, peak density, and infection duration between reference data and simulation with calibrated immunity and gametocyte development parameters. [file 12936_2015_751_MOESM3_ESM.pdf]

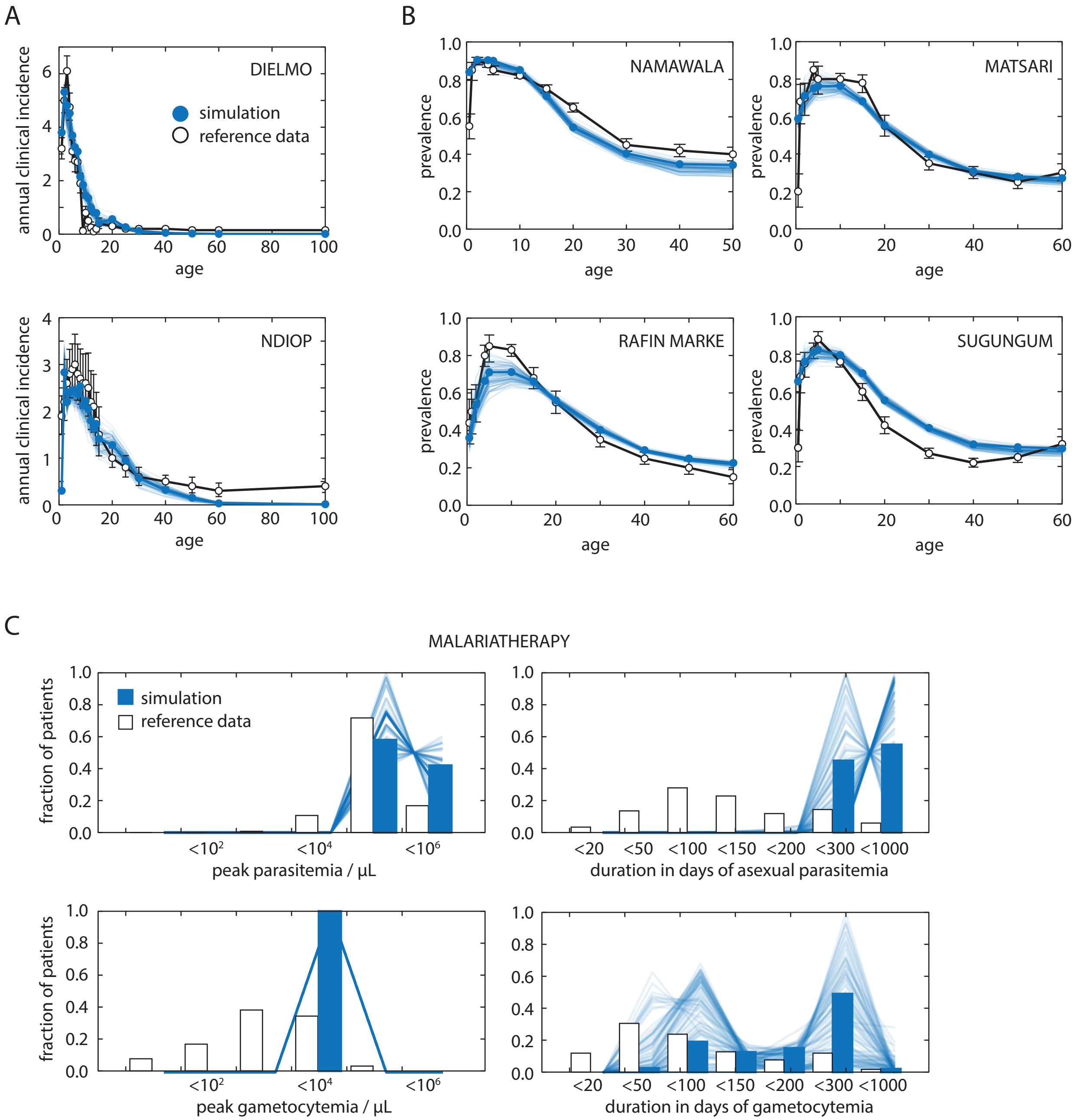


Comparison of incidence, prevalence, peak density, and infection duration between reference data and simulation with calibrated immunity and gametocyte development parameters. Simulation data corresponds to the single (solid blue) and 100 (transparent blue) parameter set(s) with highest combined likelihood across all study sites.
