# Supplementary material for: Characterization of the infectious reservoir of malaria with an agent-based model calibrated to age-stratified parasite densities and infectiousness
Source: Malar J. 2015 Jun 3;14:231. doi: 10.1186/s12936-015-0751-y (PMC4702301; doi:10.1186/s12936-015-0751-y)
Supplement: Additional file 4: — Comparison between simulation and reference data after calibration of immune and gametocyte parameters with full weighting of malariatherapy data. [file 12936_2015_751_MOESM4_ESM.pdf]

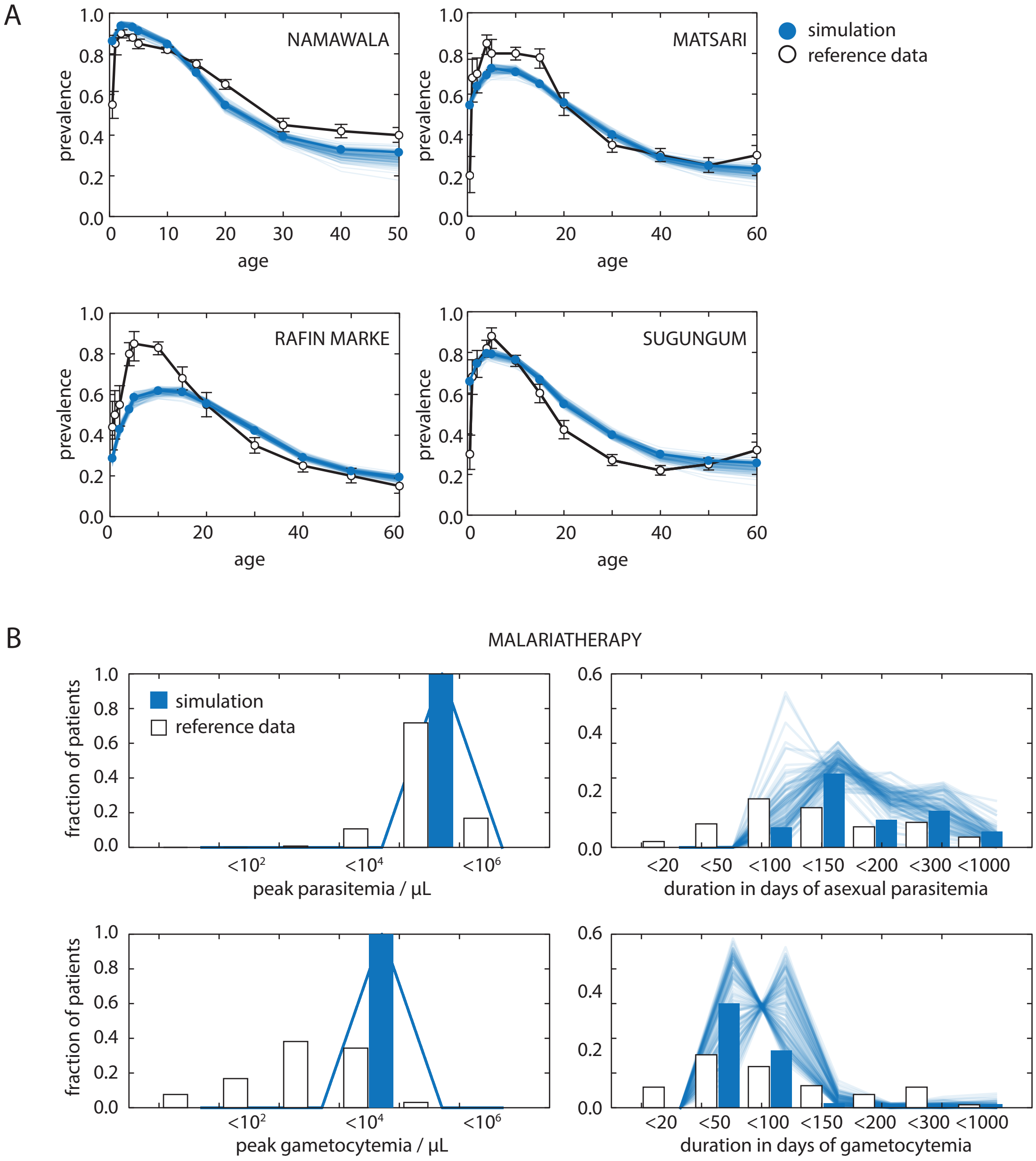

Comparison between simulation and reference data after calibration of immune and gametocyte parameters with full weighting of malariatherapy data. Fit to prevalence data is poorer than in Figure S1.
