# Supplementary material for: Characterization of the infectious reservoir of malaria with an agent-based model calibrated to age-stratified parasite densities and infectiousness
Source: Malar J. 2015 Jun 3;14:231. doi: 10.1186/s12936-015-0751-y (PMC4702301; doi:10.1186/s12936-015-0751-y)
Supplement: Additional file 5: — Apparent EIRs tuned by scaling available larval habitat. [file 12936_2015_751_MOESM5_ESM.docx]

Apparent EIRs tuned by scaling available larval habitat

| Larval habitat multiplier | EIR under no interventions | EIR under case management | EIR with ITNs | EIR with case management and ITNs |
| --- | --- | --- | --- | --- |
| 1.0 | 221 | 201 | 64 | 37 |
| 0.6 | 145 | 128 | 24 | 9.7 |
| 0.4 | 103 | 88 | 8.7 | 3.4 |
| 0.2 | 58 | 44 | 1.5 | 0.57 |
| 0.1 | 27 | 16 | 0.42 | 0.072 |
| 0.08 | 16 | 9.0 | 0.11 | 0.0095 |
| 0.06 | 9.3 | 4.2 | 0.04 | 0.0051 |
| 0.04 | 5.5 | 2.1 | 0.02 | 0.0015 |
| 0.015 | 1.7 | 0.17 | 0.002 | 0.00031 |
| 0.010 | 0.38 | 0.008 | 0.0001 | 0.00005 |
| 0.008 | 0.05 | 0.0003 | 0.00005 | 0.00001 |
