# Supplementary material for: Characterization of the infectious reservoir of malaria with an agent-based model calibrated to age-stratified parasite densities and infectiousness
Source: Malar J. 2015 Jun 3;14:231. doi: 10.1186/s12936-015-0751-y (PMC4702301; doi:10.1186/s12936-015-0751-y)
Supplement: Additional file 6: — Sensitivity of the composition of the infectious reservoir to stochastic variation and uncertainty in parameter values for high- and moderate-transmission settings by age group and asexual parasite detection limit. [file 12936_2015_751_MOESM6_ESM.pdf]

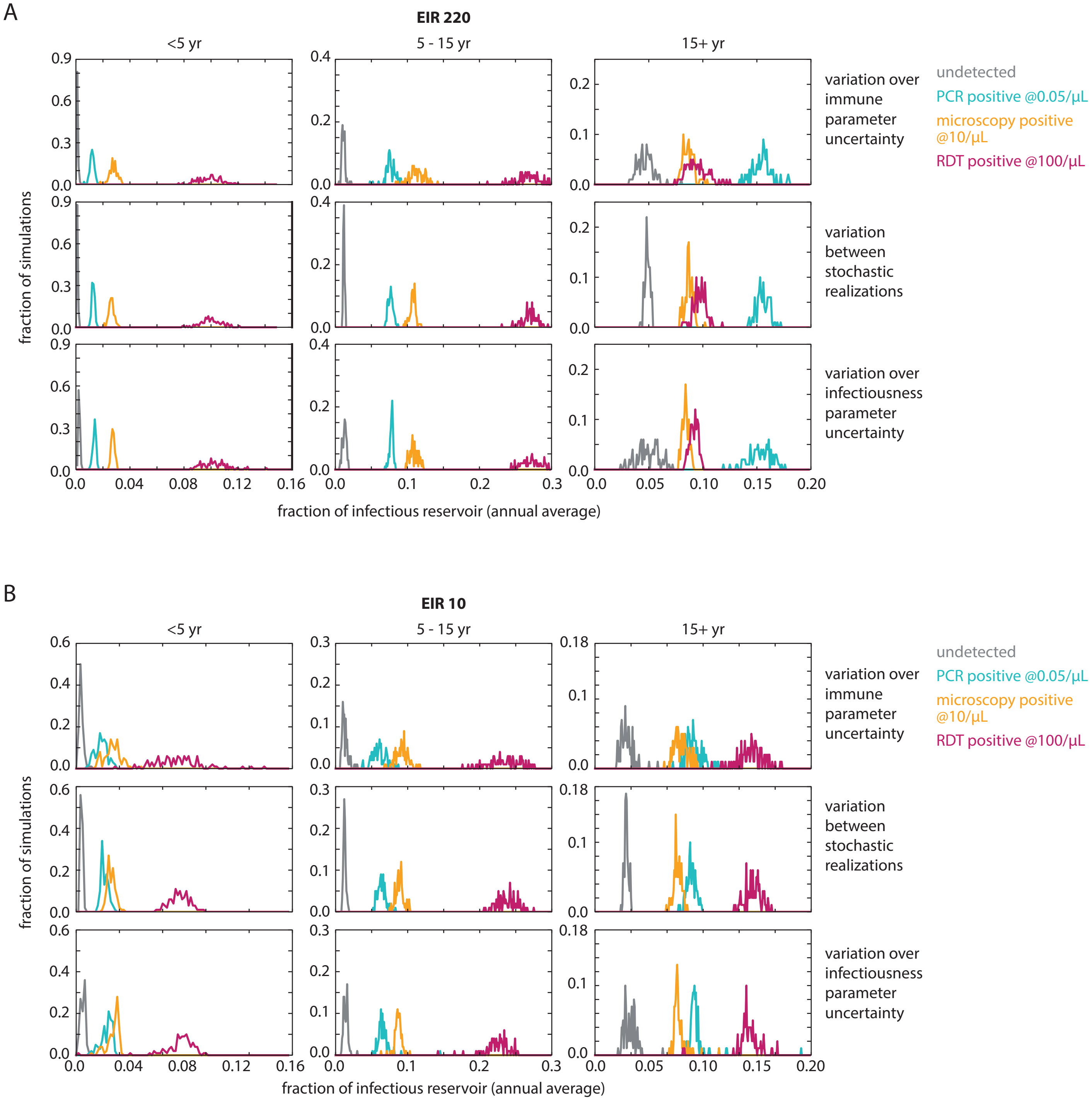

Sensitivity of the composition of the infectious reservoir to stochastic variation and uncertainty in parameter values for (A) high and (B) moderate transmission settings by age group and asexual parasite detection limit. In each row, the means of all distributions (4 detection limits x 3 age groups = 12 distributions) sum to approximately 1. Stochastic variation: 100 realizations of parameters used in Figures 1-5. Parameter uncertainties: 100 highest likelihood parameters from calibrations, 1 stochastic realization per parameter set.
