# Supplementary material for: Characterization of the infectious reservoir of malaria with an agent-based model calibrated to age-stratified parasite densities and infectiousness
Source: Malar J. 2015 Jun 3;14:231. doi: 10.1186/s12936-015-0751-y (PMC4702301; doi:10.1186/s12936-015-0751-y)
Supplement: Additional file 7: — Trajectories of infection for children and adults in high- and low-transmission settings with calibrated immune and gametocyte parameters. [file 12936_2015_751_MOESM7_ESM.pdf]

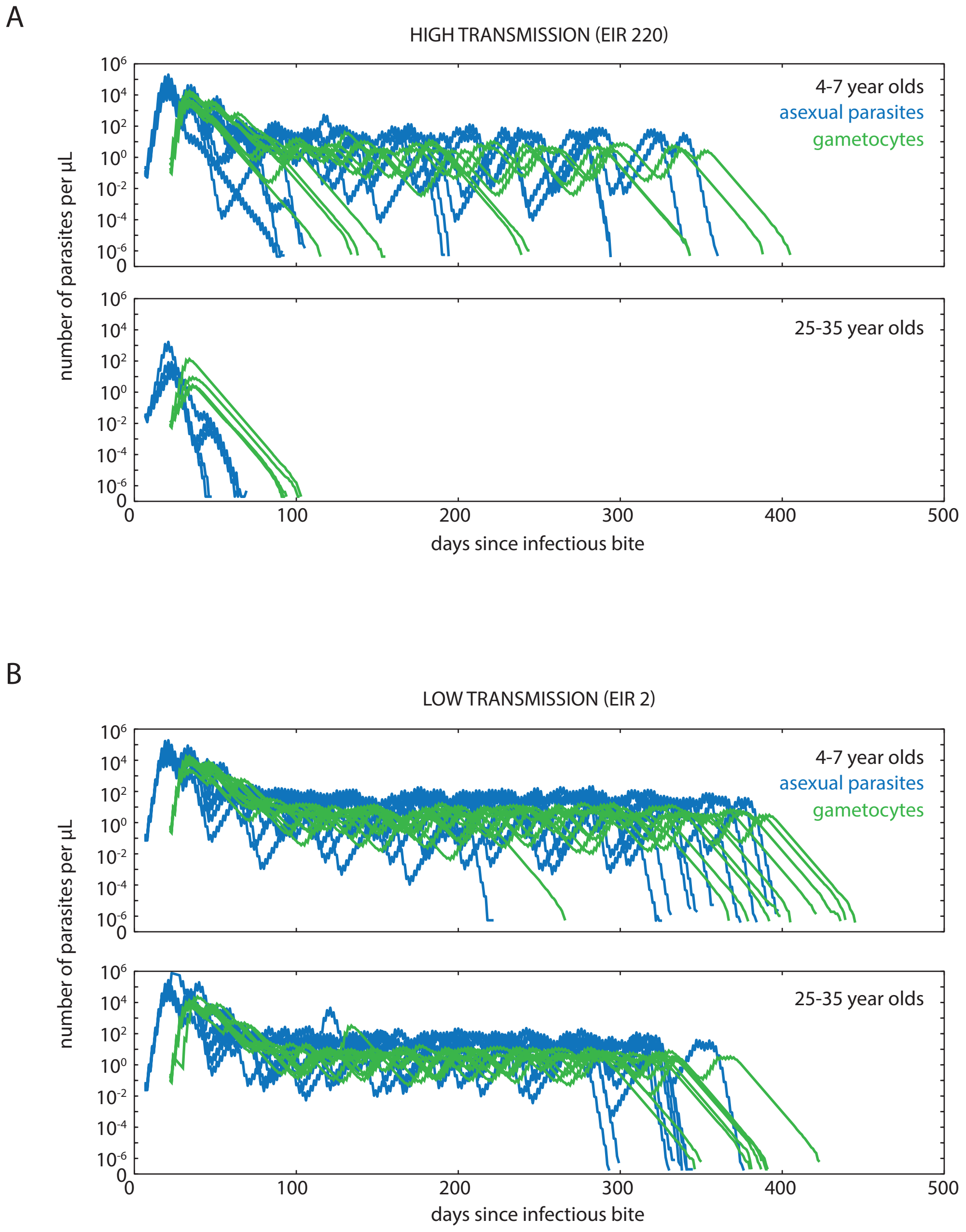

Trajectories of infection for children and adults in (A) high and (B) low transmission settings with calibrated immune and gametocyte parameters. 10 trajectories are shown for each age group. Each individual was challenged with an infectious bite on day 0 without the possibility of additional later infections.
