# Supplementary material for: Characterization of the infectious reservoir of malaria with an agent-based model calibrated to age-stratified parasite densities and infectiousness
Source: Malar J. 2015 Jun 3;14:231. doi: 10.1186/s12936-015-0751-y (PMC4702301; doi:10.1186/s12936-015-0751-y)
Supplement: Additional file 8: — Unscaled total infectiousness by diagnostic threshold and age group. [file 12936_2015_751_MOESM8_ESM.pdf]

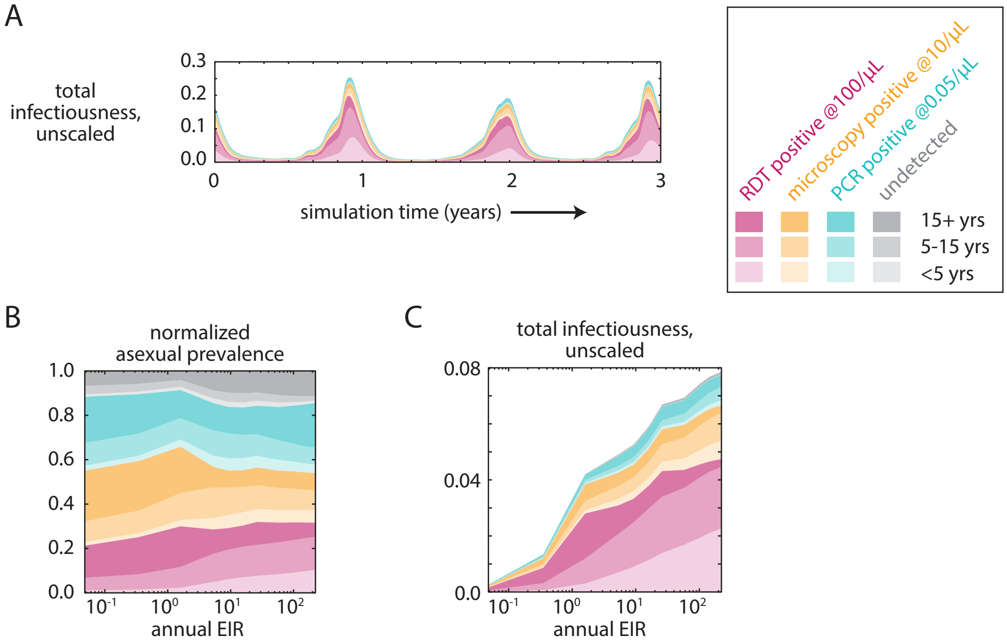

Unscaled total infectiousness by diagnostic threshold and age group. The unscaled total infectiousness is the fraction of mosquitoes that would be infected if the same number of mosquitoes fed on each person in the population. (A) Unscaled total infectiousness over 3 simulation years for setting with EIR = 10. (B) Normalized annual average asexual parasite prevalence over a range of EIRs. (C) Annual average total infectiousness over a range of EIRs.
