# Supplementary material for: Characterization of the infectious reservoir of malaria with an agent-based model calibrated to age-stratified parasite densities and infectiousness
Source: Malar J. 2015 Jun 3;14:231. doi: 10.1186/s12936-015-0751-y (PMC4702301; doi:10.1186/s12936-015-0751-y)
Supplement: Additional file 9: — Malaria transmission under case management and ITN use. [file 12936_2015_751_MOESM9_ESM.pdf]

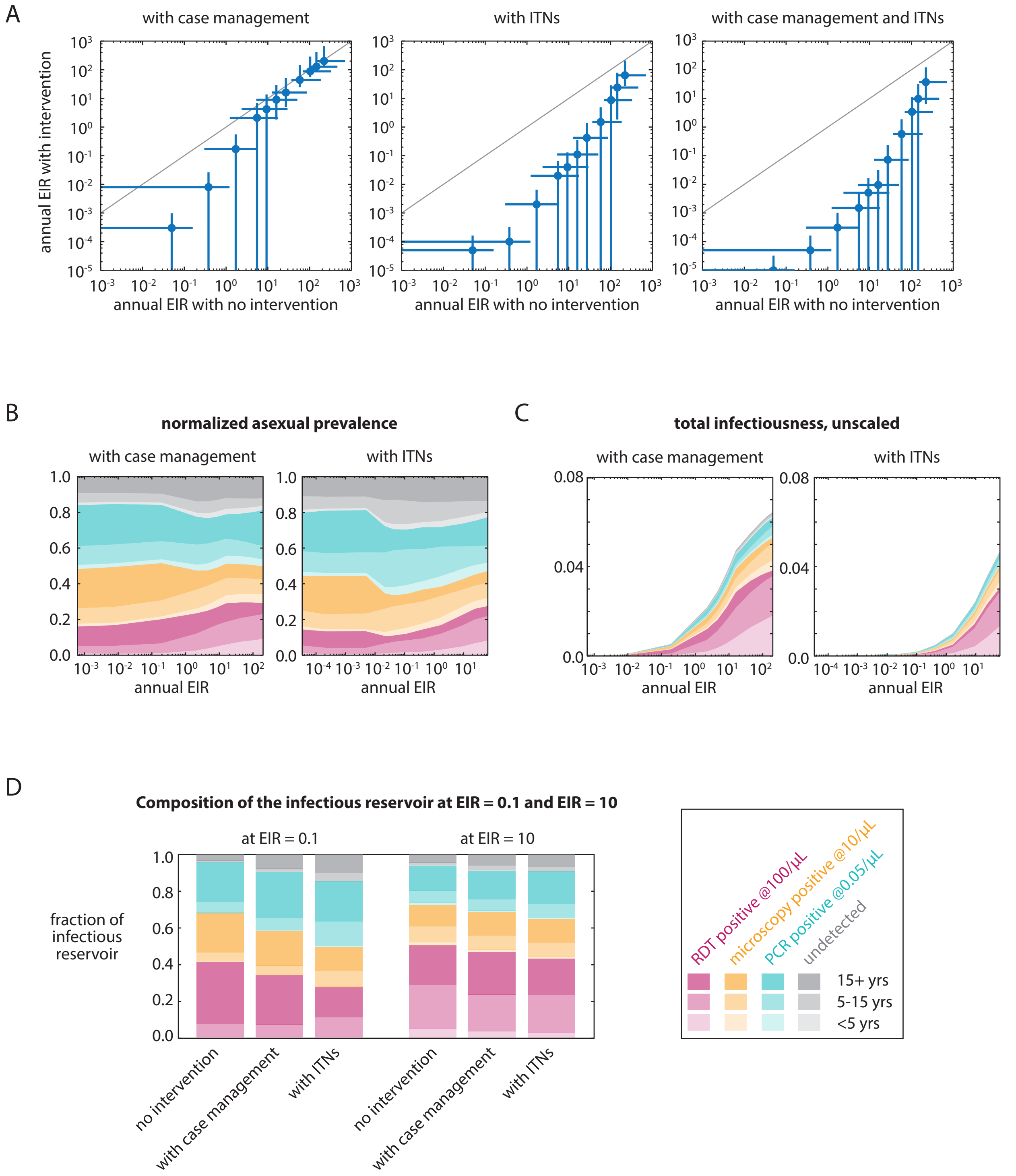

Malaria transmission under case management and ITN use.
(A) Apparent annual EIR drops with case management and ITN campaigns. Diagonals indicate locations of no change in apparent EIR upon addition of interventions.
(B) Normalized annual average asexual parasite prevalence under case management and ITN use.
(C) Annual average unscaled total infectiousness under case management and ITN use.
(D) Comparison of the infectious reservoir at apparent EIR of 0.1 and 10 for settings with no interventions, case management alone, and ITN use alone.
